# Targeting Renin–Angiotensin System Against Alzheimer’s Disease

**DOI:** 10.3389/fphar.2018.00440

**Published:** 2018-04-30

**Authors:** Abadi Kahsu Gebre, Birhanetensay Masresha Altaye, Tesfay Mehari Atey, Kald Beshir Tuem, Derbew Fikadu Berhe

**Affiliations:** ^1^Department of Pharmacology and Toxicology, School of Pharmacy, College of Health Sciences, Mekelle University, Mekelle, Ethiopia; ^2^Clinical Pharmacy Unit, School of Pharmacy, College of Health Sciences, Mekelle University, Mekelle, Ethiopia

**Keywords:** RAS, ARB, ACEI, amyloid β, oxidative stress, vascular disease, inflammation, AD

## Abstract

Renin Angiotensin System (RAS) is a hormonal system that regulates blood pressure and fluid balance through a coordinated action of renal, cardiovascular, and central nervous systems. In addition to its hemodynamic regulatory role, RAS involves in many brain activities, including memory acquisition and consolidation. This review has summarized the involvement of RAS in the pathology of Alzheimer’s disease (AD), and the outcomes of treatment with RAS inhibitors. We have discussed the effect of brain RAS in the amyloid plaque (Aβ) deposition, oxidative stress, neuroinflammation, and vascular pathology which are directly and indirectly associated with AD. Angiotensin II (AngII) via AT1 receptor is reported to increase brain Aβ level via different mechanisms including increasing amyloid precursor protein (APP) mRNA, β-secretase activity, and presenilin expression. Similarly, it was associated with tau phosphorylation, and reactive oxygen species generation. However, these effects are counterbalanced by Ang II mediated AT2 signaling. The protective effect observed with angiotensin receptor blockers (ARBs) and angiotensin converting enzyme inhibitors (ACEIs) could be as the result of inhibition of Ang II signaling. ARBs also offer additional benefit by shifting the effect of Ang II toward AT2 receptor. To conclude, targeting RAS in the brain may benefit patients with AD though it still requires further in depth understanding.

## Introduction

Renin Angiotensin System (RAS) is a hormonal system that regulates body fluid, electrolyte homostasis, and vascular tone (Yim and Yoo, 2008; Sparks et al., 2014). These classical functions of RAS are mediated by angiotensin effector peptides including Ang II, III and 1–7 (Atlas, 2007). Ang II, the primary effector peptide, is produced in the blood and exerts a number of effects on kidney, adrenal glands, sympathetic nervous system and baroreceptor reflexes (Reid, 1992; Dasgupta and Zhang, 2011). Studies have also shown the presence of local RAS in many different tissues including brain (Ganten et al., 1983; Wang et al., 1996; Vila-Porcile and Corvol, 1998; Grobe et al., 2010; Ferrão et al., 2014). In the central nervous system, angiotensinogen is synthesized by astrocytes and subsequently cleaved by renin, angiotensin converting enzyme (ACE) and aminopeptidases or ACE2 and Neprilysin (Bodiga and Bodiga, 2013). Despite some speculations, it is not clearly known where these RAS enzymes are locally synthesized in the brain (McKinley et al., 2003).

The angiotensin ligands interact with their receptors including angiotensin (AT) 1A, 1B, 2, 4 and Mas and controls various brain function (Guimond and Gallo-Payet, 2012; Premer et al., 2013). The receptors are differentially expressed in several parts of the brain (Kakar et al., 1992; Braga, 2011). AT1A is expressed in areas mainly involved in regulation of blood pressure and electrolyte balance including subfornical organ, paraventricular nucleus of the hypothalamus, lateral septum, cerebral cortex, and hippocampus (Johren et al., 1995; MacGregor et al., 1995; Lenkei et al., 1997), brainstem baroreflex arc, olivocerebellary system, and preoptic region (Lenkei et al., 1997). While AT1B is expressed in structures which involve in higher brain function and memory including cerebral cortex and hippocampus (Johren et al., 1995).

Activation of AT1 receptors is associated with increase in oxidative stress (Prusty et al., 2017), anxiety and stress (Saavedra et al., 2005; Wincewicz and Braszko, 2014), ischemic brain damage (Panahpour et al., 2014), and cognitive impairment (Nakagawa et al., 2017).

AT2 receptor, on the other hand, is observed in parts of the brain which regulate learning and memory including hippocampus, cingulate cortex, superior colliculus, lateral septum, in thalamic nuclei, in the subthalamic nucleus, in the locus coeruleus, and in the inferior olive (Millan et al., 1991; Lenkei et al., 1996). AT2 receptor is also expressed in brain structures including red nucleus, pedunculopontine tegmental nucleus, bed nucleus of the supraoptic decussation, paragenual nucleus, motor hypoglossal nucleus, cerebellar nuclei (Song et al., 1991, 1992; Tsutsumi and Saavedra, 1991; Lenkei et al., 1996), substantia nigra (Garrido-Gil et al., 2013; Valenzuela et al., 2016), and ventral tegmental area (Garrido-Gil et al., 2013). However, the extent of the receptor expression is limited after the fetal period (AbdAlla et al., 2009). AT2 receptor signaling is suggested to play beneficial role in neurogenesis (Umschweif et al., 2014), cerebral blood flow (Iwai et al., 2004; Fuchtemeier et al., 2015), neuronal plasticity (Namsolleck et al., 2013), and learning and memory (Jing et al., 2012). Activation of the receptor is also reported to attenuate inflammation (Rompe et al., 2010), oxidative stress (Lu et al., 2015) and abnormal neuronal firing (Grammatopoulos et al., 2004; Matsuura et al., 2005) observed as the result of AT1 receptor stimulation (Guimond and Gallo-Payet, 2012).

In addition to AT1 and AT2 receptors, recent evidences show the presence of other receptors in CNS including AT4 and Mas (Singh and Karnik, 2016). AT4 receptor interacts with a different angiotensin ligand called angiotensin IV, and it is reported to regulate learning and memory in brain areas including the hippocampus, neocortex and motor nuclei (Wright et al., 1999; Chai et al., 2000). The receptor is also localized in claustrum, choroid plexus, pontine nucleus, thalamic nuclei, substantia nigra pars compacta and hypothalamus (Zhuo et al., 1998; Chai et al., 2000). It is also suggested for its neuroprotective effect against cerebral ischemia (Faure et al., 2006). Mas receptor also contributes for the diverse actions of RAS in the brain (Jackson et al., 2018). The receptor is mainly localized in the hippocampus, amygdala, anterodorsal thalamic nucleus, cortex, and hypoglossal nucleus (Bunnemann et al., 1990; Becker et al., 2007; Freund et al., 2012; Lazaroni et al., 2012). Activation of the receptor by angiotensin 1–7 was found to strengthen synapses in areas involved in memory (Bunnemann et al., 1990; Hellner et al., 2005; Uekawa et al., 2016).

Brain RAS generally involves in regulating central activities including learning, memory, anxiety, depression, cognition, and emotional stress (Gard, 2004; Paul et al., 2006; de Gasparo et al., 2013), but it also complements functions of the peripheral RAS (McKinley et al., 2003). Importantly, there are growing evidence indicating the contribution of brain RAS in development of neurodegenerative disorders including AD (Zhu et al., 2011; Tian et al., 2012; AbdAlla et al., 2013; Ana Flavia et al., 2017; Takane et al., 2017). However, it is not exactly known how RAS system influences the development and progression of AD. It is not also well understood how medications acting on RAS system affect AD though some studies have shown a link between RAS and accumulation of toxic Aβ peptides (Murphy and LeVine, 2010; Gouras et al., 2015), tau phosphorylation (Tian et al., 2012), oxidative stress (Chrissobolis et al., 2012), mitochondrial dysfunction (Nozoe et al., 2008), neuroinflammation (Vargas et al., 2012) and cholinergic dysfunction (Barnes et al., 1990).

## Amyloid and Alzheimer’s Disease

Aβ_42_ and Aβ_40_ are the two-predominant Aβ-proteins that are highly susceptible for aggregation to form oligomers, protofibrils, and fibrils (Shin et al., 1997; Ahmed et al., 2010). Under normal physiological conditions, brain eliminates toxic peptides via enzymatic degradation, perivascular drainage and receptor-mediated efflux transport (Higuchi et al., 2005; Wang et al., 2011; Iliff et al., 2012; Provias and Jeynes, 2014; Baranello et al., 2015). Impairment of either of these clearance mechanisms may result in accumulation of Aβ peptide. The accumulation can cause neuronal membrane damage, an increase in oxidative stress, receptor-mediated alteration of signal transduction, alteration of membrane pore, increase in intracellular level of calcium ion and mitochondrial damage (Yankner, 1996; Carrillo-Mora et al., 2014). These changes also trigger persistent loss of cholinergic projections to the neocortex (Supnet and Bezprozvanny, 2010).

Aβ deposition facilitates the formation of pathological phosphorylated tau proteins (Busciglio et al., 1995; Zheng et al., 2002; Bloom, 2014). Accumulation of toxic tau protein could also occur independent of amyloid β (Katsuno et al., 2005). The abnormal aggregation and deposition of tau protein can result in formation of neurofibrillary tangles leading to a progressive loss of neurons (Buee et al., 2000; Hanger et al., 2009; Wolfe, 2012). Tau mediated neurodegeneration could be due to sequestration of tau protein and disturbance of microtubule function (Alonso et al., 2008; Iqbal et al., 2009). This results impairment of normal axon flow and subsequent loss of neurons and their connectivity (Iqbal et al., 2009; Baird and Bennett, 2013).

## Renin Angiotensin System and Aβ Peptides: *In Vitro* Studies

*In vitro* studies have shown the role of ACE in the degradation of Aβ peptides halting the halts development of amyloid plaque (Hu et al., 2001; Oba et al., 2005). The enzymatic action of ACE in the breakdown Aβ peptides have demonstrated by several studies (Hemming and Selkoe, 2005; Sun et al., 2008; Zou et al., 2009). Whilst ACE inhibitors were reported to promote Aβ aggregation (Hu et al., 2001). ACE2, a homolog of ACE, was also reported to have a catalytic role in the cleavage of Aβ_43_ to Aβ_40_ and this was inhibited by specific ACE2 inhibitor called DX600 (Liu et al., 2014). N domain part of the enzyme was found responsible for hydrolysis Aβ peptides at N-terminal position. ACE hydrolyses the most neurotoxic peptides Aβ_43_ and Aβ_42_ (Welander et al., 2009; Brouillette et al., 2012), in to amyloid peptides that are less susceptibility to aggregate and form senile plaques. ACE also metabolizes the most abundant amyloid peptide, Aβ_40_ with the potential to reduce the Aβ_42_ oligomerization and deposition (Kim et al., 2007; Murray et al., 2009). ACE reduces amyloid β peptides the main risk factor for the development and progression of AD (Karran et al., 2011) (**Table 1**). These studies altogether indicate the metabolic action of RAS enzymes in reducing amyloid plaque deposition via degradation of the most toxic form amyloid peptides composed of 40-43 amino acid sequences.

**Table 1:** The effect of ACE-Is on Amyloid-β level: *In vitro* study.

Cloned culture	Effects of ACE expression	Effects of ACE inhibition	Reference
Seminal plasma	Decrease Aβ_40_ level	Lisinopril promote Aβ_40_ production	Hu e/al., 2001
Neuroblastoma	Decrease Aβ_40_ and Aβ_42_ level	Captopril promote Aβ_40_ and Aβ_42_ level	Hemming and Selkoe, 2005
HEK293	Increase break down of Aβ_43_ to Aβ_42_	DX600 inhibit breakdown Aβ_43_ to Aβ_42_	Liu et al., 2014
COS7 cells	Increase breakdown of Aβ_40_	**–**	Oba et al., 2005
CHO cells	Increase breakdown of m and h Aβ	**–**	Sun et al., 2008
COS7 cells	Increase breakdown of Aβ_42_ to Aβ_40_	ACEIs inhibit conversion of Aβ_42_ to-Aβ_40_	Zou et al., 2009


**ACEIs, angiotensin converting enzyme inhibitors; CHO, Chinese hamster ovary; HEK, human embryonic kidney cells 293; Aβ, Amyloid-β; m Aβ, murine Amyloid- β; h Aβ, human amyloid- β.**

## Renin Angiotensin System and Alzheimer’s Disease: Animal Studies

*In vitro* studies have shown the role of ACE in degradation of Aβ peptides thereby reducing deposition and accumulation of amyloid plaque while inhibition of the enzyme is detrimental (Hemming and Selkoe, 2005; Sun et al., 2008; Zou et al., 2009; Liu et al., 2014). Ramipril (ACE inhibitor) also increased Aβ peptides in ACE10/10 mice with AD (Bernstein et al., 2014). Recent studies, however, does not support the idea that ACEIs increases accumulation of Aβ peptides in AD animal models (Eckman et al., 2006; Hemming et al., 2007; Ferrington et al., 2011, 2012). These studies challenge the notion that ACEIs inhibit degradation of Aβ peptides and favoring amyloid plaque formation. Some ACEIs even reduced Aβ peptide level in animal models of AD (AbdAlla et al., 2013). Moreover, ACEIs showed beneficial effect in reducing AD signs and symptoms (Dong et al., 2011; Tota et al., 2012; AbdAlla et al., 2015). Administration of perindopril (ACEI) has shown an instrumental effect in increasing density of normal neurons and improving learning and memory (Hou et al., 2008). A study on Tg2576 AD model demonstrated the positive role of captopril in preventing signs of neurodegeneration (AbdAlla et al., 2013). These studies support the potential benefit of ACEIs in alleviating sign and symptom of AD; however, with contrasting reports. A study on Tg2576 mice showed increase in deposition of Aβ_42_ after treatment with captopril (Zou et al., 2007). In line with this study, treatment with ramipril elevated brain level of Aβ_42_ peptide in AD^+^ACE (10/10) mice. Most *in vivo* studies have shown a positive correlation between increased expression of ACE and signs of AD but ACE inhibitors have protective effect against AD (**Table 2**). The protective effect of ACE inhibitors could be explained partly via suppressing brain derived neurotrophic factor decline and TNF-α release. They were also found to ameliorate oxidonitrosative stress and nitrotyrosine production (Ali et al., 2016) with that in turn reduces amyloidogenesis and subsequent Aβ deposition (Goel et al., 2016). However, further investigations are required to see if the contradicting reports were intrinsic to the specific inherent nature of the drug or methodological issue.

**Table 2:** The effect of ACEIs on Brain A level: Animal studies.

Animal model	Tested drug	Results	Reference
Aβ_42_ induced SDR	Perindopril	Decrease Aβ_42_	Hou et al., 2008
Tg2576 mice	Captopril/Enalapril	Reduced Aβ plaque and ROS accumulation	AbdAlla et al., 2013
C57BL/6 × DBA2 and 3xTg AD	Captopril	No effect on Aβ levels	Hemming et al., 2007
LPS induced Mice	Perindopril	Decrease Aβ level	Ali et al., 2016
LPS induced WRs	Perindopril	Decrease Aβ levels and improved CBF	Goel et al., 2016
A E10/10 mice	Ramipril	Increase Aβ levels	Bernstein et al., 2014


**ACE, angiotensin converting enzyme; LPS, lipopolysaccharide; ROS, reactive oxygen species*.*

A review by Kehoe indicated Ang II (as with ACE) increased accumulation and deposition Aβ peptides in AD animal models (Kehoe, 2009). Ang II increases Aβ level, promotes cerebrovascular dysfunction, and micro-vascular amyloid deposition which those in turn worsens AD outcome (Faraco et al., 2016). ARBs, e.g., telmisartan, have shown to prevent cognitive decline associated with Aβ_40_ injection (Mogi et al., 2008). Olmesartan was also associated with improved cognitive function and hippocampal synaptic plasticity (Takeda et al., 2009). Losartan was reported to prevent neuropathological and cognitive deficits observed in AD (Ongali et al., 2014). These studies showed the beneficial roles of ARBs in animal models of AD. The protective effect could be explained in part via suppressing AT1 receptor mediated APP mRNA up regulation, Aβ peptide production and phosphorylated tau induced neurotoxicity (Zhu et al., 2011). The protective effect of these drugs could also be attributed as a result of unopposed action of Ang II on AT2 receptor (Horiuchi et al., 2010; Gallo-Payet et al., 2011) and stimulation of AngIV/AT4R signaling as observed in losartan (Royea et al., 2017). AT2 receptor mediated signaling pathways are known to prevent degeneration of neurons (Li et al., 2005; McCarthy et al., 2009). In line with these reports, valsartan have shown to attenuate oligomerization of Aβ peptides into high molecular weight oligomeric peptides and reduces cognitive deterioration (Wang et al., 2007). However, other studies with the same model have shown that Aβ induces the formation of oligomers of AT2 receptor in the hippocampus that disrupts Ang II mediated signaling. The Aβ- induced AT2 receptor oligomerization was associated with enhanced neurodegeneration. Conversely, inhibition of cross-linked AT2 receptor delayed tau phosphorylation (AbdAlla et al., 2009).

**FIGURE 1:**
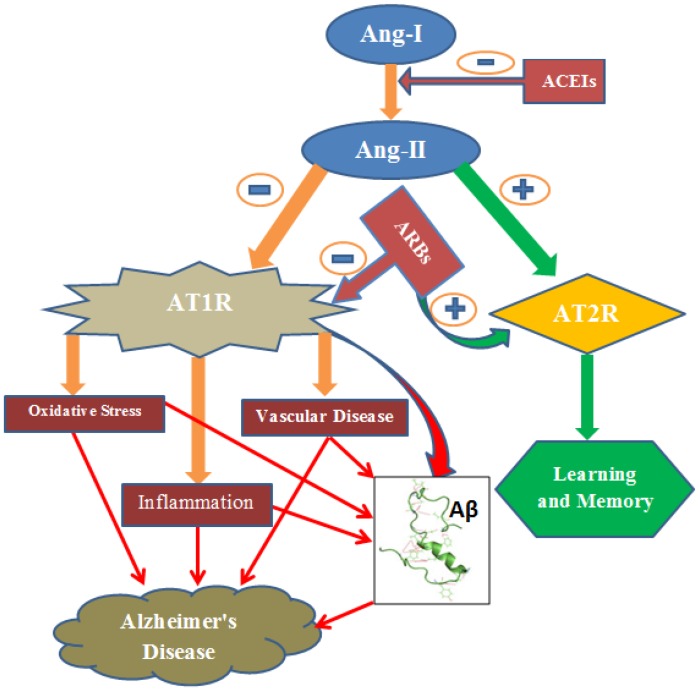
Ang-II induces oxidative stress, inflammation and vascular disease via AT1R. Consequently, it causes accumulation of amyloid-β resulting Alzheimer’s disease. However, AT2 R signaling produces beneficial effect including learning and memory. ARBs inhibit AT1R signaling and this shifts the action of Ang-II toward the beneficial pathway (AT2R signaling). ACEIs, Angiotensin converting enzyme inhibitors; ARBs, Angiotensin Receptor blockers; AT1R, Angiotensin 1 Receptor; AT2R, Angiotensin 2 Receptor; Aβ, Amyloid-β; -, negative outcome or blockage; +, positive outcome.

In other studies, however, valsartan or eprosartan (ARBs), did not alter accumulation of Aβ oligomers and phosphorylated tau in triple transgenic mice (Ferrington et al., 2011). The contradiction could be reconciled by the difference in AD animal models used. Variability in the dose of drug, the age and strain of animal used in the experiment could also explain the discrepancy (Ferrington et al., 2012). Despite varying result of RAS on amyloidosis, the overall effects of this system seem to favor amyloidosis. More specifically, the Ang II favors production of Aβ peptides via the most widely expressed angiotensin receptor, AT1 (Hohle et al., 1995). In addition to reduction of Aβ deposition and its consequences, RAS inhibitors have also other beneficial roles including suppression of inflammation (Saavedra, 2012), oxidative stress (Prusty et al., 2017), vascular damage/ischemia (Takeda and Morishita, 2017), and increase in acetylcholine release (Barnes et al., 1990) and glutamate uptake (Ruginsk et al., 2015) (**Figure 1**).

Ang II enhances AT1 receptor mediated brain inflammation. Contrarily, ARBs attenuates the release of proinflammatory mediators (Lanz et al., 2010). Central infusion of Ang II increased hippocampal CD68- positive cells, indicating its hippocampal proinflammatory action (Takane et al., 2017). In contrarily, candesartan (ARB) decreased lipopolysaccharide (LPS) induced and AT1 receptor mediated release of proinflammatory mediators including TNFα, IL-1β, IκBα, iNOS, ICAM-1, and VCAM-1 in cerebral cortex (Benicky et al., 2009). In addition, candesartan attenuated brain level of NF-α, GFAP, COX-2, and NF-κB in the same animal model. They have also demonstrated the advantage of unopposed action of Ang II on AT2 receptor in addition to AT1 receptor blockage mediated amelioration of proinflammatory mediators releasesuggesting the beneficial role of AT2 receptors in reducing neuroinflammation (Goel et al., 2018). Moreover, ARBs prevents impairment and preserves the integrity of blood brain barrier which in turn reduces infiltration of inflammatory mediators observed in many neurodegenerative disease including AD (de Vries et al., 1997; Panahpour et al., 2014; So et al., 2015).

AngII via AT1 receptor is also suggested as effector of oxidative stress (Nickenig and Harrison, 2002; Marchesi et al., 2008; Chan and Chan, 2013; Seifi et al., 2014; Prusty et al., 2017). Ang II increased a reactive oxygen species called superoxide (Takane et al., 2017). On the other hand, telmisartan (ARB) was found to normalize diminished thioredoxin (Trx) system in addition to attenuating thioredoxin-interacting protein (TXNIP) expression. This reduces generation of endogenous reactive oxygen species (Erdi et al., 2016). Similarly, telmisartan reduced advanced glycation end products and 4-hydroxynonenal, which are the markers of oxidative stress and associated with Neurodegeneration (Safciuc et al., 2007; Barone et al., 2017). Candesartan also reduced brain level of free radicals by diminishing Malondialdehyde and increasing glutathione level (Tota et al., 2009). Thus partly alleviates the development and progression of AD (Gustaw-Rothenberg et al., 2010; Saharan and Mandal, 2014). Captopril (Bild et al., 2013) and losartan (Seifi et al., 2015) were also found to ameliorate oxidative stress.

Ang II is also implicated in neurovascular damage and cognitive impairment (Mogi et al., 2012; Bodiga and Bodiga, 2013; Bloch et al., 2015). Candesartan increased cerebral blood flow, reduced infarct size and improved cerebral ischemia (Ito et al., 2002; Engelhorn et al., 2004). Similarly, losartan prevented blood brain barrier disruption and restored blood flow after induction of hemorrhagic stroke. Moreover, telmisartan (Iwanami et al., 2010), valsartan (Takada et al., 2006), and olmesartan (Matsumoto et al., 2009) have shown a beneficial role in prevention of vascular damage via blockage of AT1 receptor. Suggested mechanism of ARBs on cerebral blood flow is in part explained via unblocked AT2 receptor activation (Iwai et al., 2004; Li et al., 2005; Jing et al., 2012). These studies generally show the benefit of ARBs in improving neurovascular network and cerebral blood flow after certain initial insult which in turn prevents onset and progressive neurodegeneration observed in AD (Bell and Zlokovic, 2009; Zlokovic, 2011). In addition to the above mechanisms described, Ang II is also speculated to inhibit acetylcholine release in which the deficiency is responsible for AD (Barnes et al., 1992; Tota et al., 2013). Conversely, pre-treatment with candesartan prevented Ang II induced reduction of acetylcholine level (Tota et al., 2009, 2013). This reduces cognitive impairment observed in AD (Burns, 2003; Herholz, 2008).

## Renin Angiotensin System and Alzheimer’s Disease: Human Studies

Human studies have shown the involvement of RAS in the pathogenesis and progression of AD (Amouyel et al., 2000; Kolsch et al., 2005). Nevertheless, only few studies have shown a link between RAS and AD (Ellul et al., 2007; Davies et al., 2011). ACEIs and ARBs have shown a beneficial effect in slowing and reducing the cognitive impairment associated with AD (Li et al., 2012; Hsu et al., 2013; Saavedra, 2016). In a cross sectional study, patients taking ARBs and ACEIs had lower risk of cognitive deterioration (Jackson et al., 2018).

Central acting RAS inhibitors have shown a superior efficacy which imply brain RAS involvement in development and progression of AD (Hebert et al., 2013; Soto et al., 2013; Wharton et al., 2015; Zhuang et al., 2016). A prospective multicentre cohort study showed slower rate of cognitive decline on older adults taking ACE-Is (Soto et al., 2013). ARBs and ACEIs were generally found to reduce the risk and progression of AD (Hajjar et al., 2008; Li et al., 2010; Davies et al., 2011). The central acting agent including perindopril was significantly associated with a slower rate of functional decline (Davies et al., 2011). Telmisartan reduced cognitive impairment in hypertensive patients with AD (Li et al., 2012). The drug reduced amyloid β, oxidative stress and neuroinflammation. The RAS also activates peroxisome proliferator activated receptor (PPAR) gamma which has a role in prevention of neurodegeneration (Inestrosa et al., 2005; Kume et al., 2012; Li et al., 2012). Other ARBs losartan (Moriwaki et al., 2004; Hong et al., 2010), and olmesartan (Matsumoto et al., 2010) have shown beneficial effect in AD patients. In contrast, in a 4-month of pilot clinical trial ramipril was not associated with reduction of CSF Aβ_1-42_ level and cognitive impairment (Wharton et al., 2012). This limited effect of ramipril could be attributed to its limited blood brain barrier penetration (Sink et al., 2009). Most of these studies support the beneficial effect of RAS inhibitors in prevention and mitigation of cognitive impairments associated with AD (**Table 3**).

**Table 3:** The effect of ACEIs and ARBs on cognitive function: Human study.

Study design	Tested drug	Result	Reference
Cross sectional	ACE-Is and ARBs	Reduce cognitive decline	Ellul et al., 2007
Observational	ACEIs	Slow decline of memory and daily functions	Hajjar et al., 2008
Case Control	ACE-Is and ARBs	Decease incidence of AD	Davies et al., 2011
Cohort	ARBs	Reduction in the incidence and progression of AD	Li et al., 2010
Cohort	ACE-Is	Slow cognitive decline	Soto et al., 2013
Observational	RAS-Ms	Slows cognitive decline	Wharton et al., 2015
Observational	CACE-Is	Reduce functional decline	O’Caoimh et al., 2014
Cohort	ACEIs	Not effect on cognitive decline	Zhuang et al., 2016


**ACEIs, angiotensin converting enzyme inhibitors; ARBs, angiotensin II Receptor Blockers; RAS-Ms, renin angiotensin system medications; CACE-I, centrally acting Angiotensin converting enzyme inhibitors.**

## Genetic Studies

Genetic studies have also reported for the associate of ACE with AD (Elkins et al., 2004). ACE protein is coded by several genes containing various variants, specifically the insertion/deletion variant (rs1799752) have been associated with AD. Some other variants, including single nucleotide polymorphisms rs4291A > T located 240 base pair from the initiation codon, and rs4343G > A encoding a silent mutation in exon 16 were also thought to be involved in AD (Helbecque et al., 2009; Gaiteri et al., 2016). AD patients with the haplotype of rs1800764 (CC): rs4291 (TT) responded better for ACEIs that can cross the blood brain barrier (captopril or perindopril). However, the response was not significant among independent carriers of rs1800764 or rs429 (de Oliveira et al., 2014). Further stratification showed the benefit of ACEIs among ACE haplotypes (rs1800764 – T and rs4291 – A) and Apolipoprotein (APOE4) – carriers (rs1800764 – T or rs4291 – T). Nevertheless, APOE4+ carriers were non-responsive for ACEIs indicating the role of genetic variation and ACEIs response rate among AD patients (de Oliveira et al., 2018).

## Conclusion

Understanding AD in terms of various pathophysiological pathways is worthwhile to unravel the complex nature of the disease process and identifying potential therapeutic targets. The brain RAS is reported to be involved in the development and progression of AD through AT1 receptor via increasing the production of amyloid-β, oxidative stress, inflammatory processes, and decreasing release of acetylcholine. However, RAS also is reported to have protective effect against AD. Through AT2 receptor activation that counterbalances the deleterious effects of AT1 receptor mediated RAS effects. With concept, beneficial effect of ARBs against AD is via the unopposed action of Ang II on AT2 receptors it as AT1 receptor is blocked these drugs increased Ang II concentration to act on AT2 receptor. ACE is reported to be involved in breakdown of amyloid β peptides, but most of the studies have contradicting result. This requires further understanding especially involvement of ACE in cleavage of amyloid β peptides *in vivo*. In summary, RAS through AT1 receptor is linked with AD pathology through its action on neurovascular change, oxidative stress, and inflammation as evidenced by the protective role of ARBs and ACEIs both in patients and animal models. However, the role of RAS in AD pathology is still not well established and need further in-depth understanding.

## Author Contributions

AG conducted the review and prepared the first draft while all authors contributed to substantial enhancement of the manuscript.

## Conflict of Interest Statement

The authors declare that the research was conducted in the absence of any commercial or financial relationships that could be construed as a potential conflict of interest.
